# Voltammetric Evaluation of Diclofenac Tablets Samples through Carbon Black-Based Electrodes

**DOI:** 10.3390/ph12020083

**Published:** 2019-06-04

**Authors:** Carlos Eduardo Peixoto da Cunha, Edson Silvio Batista Rodrigues, Morgana Fernandes Alecrim, Douglas Vieira Thomaz, Isaac Yves Lopes Macêdo, Luane Ferreira Garcia, Jerônimo Raimundo de Oliveira Neto, Emily Kussmaul Gonçalves Moreno, Nara Ballaminut, Eric de Souza Gil

**Affiliations:** Faculdade de Farmácia, Universidade Federal de Goiás, Goiânia, GO 74690-970, Brazil; cedcunhap35@hotmail.com (C.E.P.d.C.); edson.silvio.b@gmail.com (E.S.B.R.); morgana.alecrim@hotmail.com (M.F.A.); douglasvthomaz@gmail.com (D.V.T.); isaacyvesl@gmail.com (I.Y.L.M.); luane.fg@hotmail.com (L.F.G.); jeronimoneto8@gmail.com (J.R.d.O.N.); milykussmaul@gmail.com (E.K.G.M.); naraballaminut@gmail.com (N.B.)

**Keywords:** pharmaceutical electroanalysis, stability assays, anti-inflammatory drugs, pencil graphite electrodes

## Abstract

Diclofenac (DIC) is a non-steroidal anti-inflammatory drug of wide use around the world. Electroanalytical methods display a high analytical potential for application in pharmaceutical samples but the drawbacks concerning electrode fouling and reproducibility are of major concern. Henceforth, the aim of this work was to propose the use of alternative low-cost carbon black (CB) and ionic liquid (IL) matrix to modify the surface of pencil graphite electrodes (PGE) in order to quantify DIC in raw materials, intermediates, and final products, as well as in stability assays of tablets. The proposed method using CB+IL/PGE displayed good recovery (99.4%) as well as limits of detection (LOD) of 0.08 µmol L-1 and limits of quantification (LOQ) of 0.28 µmol L^−1^. CB+IL/PGE response was five times greater than the unmodified PGE. CB+IL-PGE stands as an interesting alternative for DIC assessment in different pharmaceutical samples.

## 1. Introduction

Inflammatory diseases constitute a complex and heterogeneous group of diseases, which are an important cause of disability. The pharmacological treatment of such diseases is mostly based on the use of corticosteroids and non-steroidal anti-inflammatory drugs (NSAIDs) [1,2,3]. Diclofenac (DIC) is a non-selective cyclooxygenase inhibitor belonging to NSAIDs, which acts by inhibiting the synthesis of prostaglandins that have a wide capacity to induce inflammation [1].

Pharmacopoeial methods for DIC assessment are often based in high-performance liquid chromatography (HPLC) [2,3,4], however, such techniques involve a number of pre-preparative steps, elevated cost of laboratory equipment, and are reagent-consuming methods. Although methods such as titulometry and spectrophotometry are also largely applied for DIC assessment to avoid costly methods, they lack nevertheless suitable precision and accuracy [4,5,6].

Concerning cost/benefit and environmental issues, electroanalysis emerged as a promising alternative to traditional analytical tools due to electrode versatility, selectivity, low cost, and minimal solvent use [5,6,7,8]. Amongst the myriad of electrode matrixes employed in electroanalysis, glassy carbon (GC) and carbon paste (CP) electrodes (E) are the most employed [7,8,9,10,11,12,13]; however, surface adsorption of oxidized/reduced compounds may promote electrode fouling; therefore, leading to reduced reproducibility. To overcome such drawback, electrode polishing of GCE or surface renewal of CPE are routine steps in electroanalytical assays [9,10,11,12]. In this context, pencil graphite electrode (PGE) is a soft and very cheap material whose surface layers are easily renewed by abrasion on paper [11,12,13,14,15].

Another promising and cheap approach is the use of carbon black (CB), a nanostructured material which offers the possibility to increase the effective electrode surface area. CB is a material that represents an excellent modification tool due to its enhancing qualities regarding high electrical conductivity and fast charge transfer kinetics. These characteristics are highly demanded in fields such as electrode modification and biosensors development [16]. In recent years, many applications for modifications with CB have appeared in literature, such as the modification of DNA biosensors [16], plastic films [17,18], and nano-film electrosensors [19,20,21,22].

These electrode surface modifications can be further enhanced through ionic liquid (IL) based matrices, whose intrinsic conducting properties may substantially increase method sensibility [20] without drawbacks concerning increased cost [11,12,13,14,15,16]. Moreover, IL provides anchorage to CB surface modifications, turning it feasible without further treatments.

In view of electrochemical versatility and benefits towards drug assessment, this research aimed to study the use of different electrodes, namely PGE, IL/PGE, and IL+CB/PGE as working electrodes for DIC assessment in pharmaceutical samples. The procedures herein used to renew and to improve the electroactive surface area were cleaner and easier than those applied for CPE and GCE, thus being in accordance with pharmaceutical regulatory issues.

## 2. Results and Discussions

### 2.1. Evaluation of PH Effects on Analytical Performance

**Figure 1:**
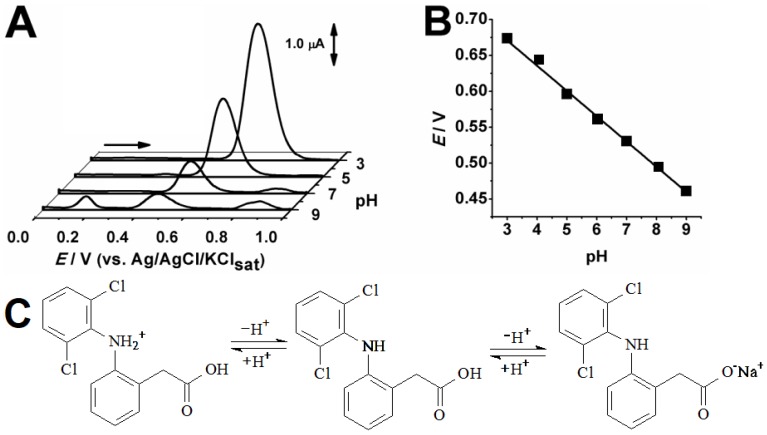
(**A**) 3D plot obtained for differential pulse voltammetric (DPV) assays at different pH solutions for 25 µmol L^−1^ Diclofenac (DIC); inset: the plot of peak current values vs. pH. (**B**) Linear plot of peak potential vs. pH unit and (**C**) the major ionic forms of DIC.

The protonation mechanisms can affect redox processes and the experimental optimization was prior assessed by differential pulse voltammetric (DPV) assays at PGE in different pH conditions. The highest peak current was found in pH 3.0 (Figure 1A), whereas the linear shift observed in the plotted *E*_pa_ vs. pH value (Figure 1B), in which the slope close to the nerstian value of 59 mV/pH shows that the proton transfer has equal participation on this redox process. In turn, the appearance of new peaks observed only in higher pH may be explained by means of electrochemical oxidation of hydrolytic products.

### 2.2. Electrochemical Behavior of DIC in PGE

**Figure 2:**
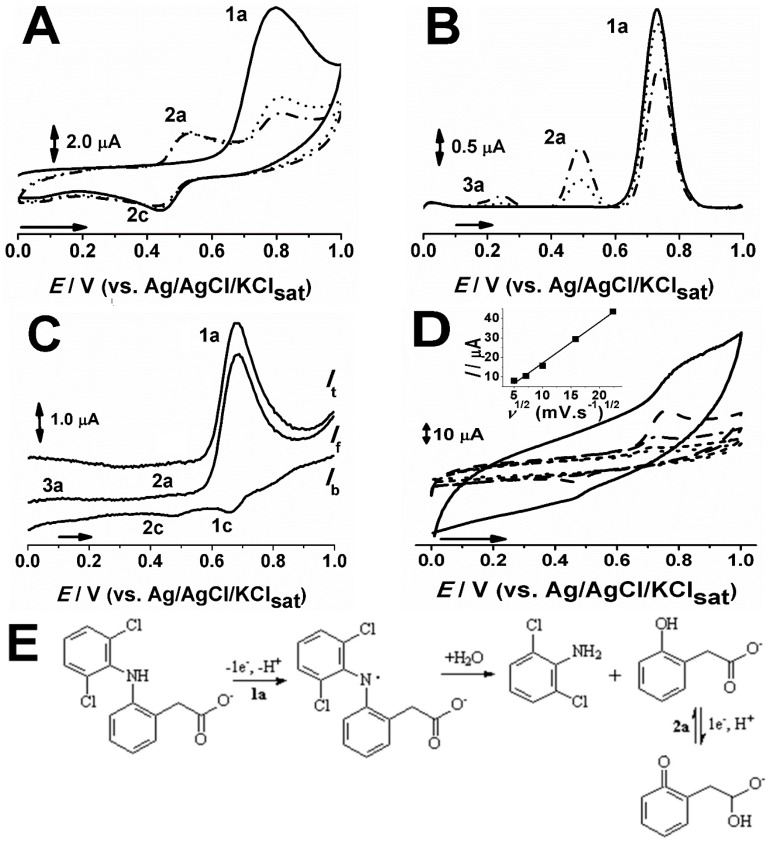
First (–––), second (• • •), and third (– • –) sequential cyclic voltammetry (CV) (**A**); DPV scans obtained at pencil graphite electrodes (PGE) without polishment prior to each assay (**B**); Square wave (SW) voltammograms, *I*_t_ as total current, *I*_f_ as forward current, and *I*_b_ as backward current (**C**); CV assays performed at different scan rates: 25 mV s^−1^ (• • •), 50 mV s^−1^ (- - -), 100 mV s^−1^ (– • –), 250 mV s^−1^ (– – –), 500 mV s^−1^ (–––) (Inset: linear plot of peak current vs. v^1/2^) (**D**). All for 25 µmol L^−1^ DIC in pH 5.0 0.1 M acetate (ACS) at PGE. The proposed electrochemical reaction mechanisms for DIC (**E**).

Figure 2A presents the sequential scans of 25 µmol L^−1^ DIC in pH 5.0 0.1 M acetate (ACS) at PGE. The first direct scan displays an anodic peak, 1a, at *E*_1a_ c.a. 0.8 V, on the reverse scan, a cathodic peak, 2c, is seen, whereas its related anodic peak, 2a, at *E*_2a_ c.a. 0.55 V, appears on the second scan as a consequence of the first anodic process (Figure 2A). These results are supported by literature data, in which the first redox process, 1a, was attributed to amine oxidation, leading to the subsequent chemical reaction [15]. In turn, a residual anodic process, 3a, seen at DPV (Figure 2B) might be attributed to para-quinonic groups electro-generated at the graphite surface [23].

Indeed, this residual process was not observed in CV assays, whereas the cathodic peak related to the anodic, 2a, can also be observed in Figure 2C. Moreover, the reversibility of redox pair 2a/2c is stated by the ratio between anodic/cathodic current signals equals to one unit, as well as, by the narrow difference between the overpotential values ~60 mV. The square wave voltammetry (SWV) assay demonstrated moreover that the first anodic process, 1a, is irreversible (Figure 2C).

In order to see the effect of scan rate on DIC anodic process, 1a, different scan rates CV assays were performed (Figure 2D). It was observed linearity (r = 0.99) between the square root of the scan rates (v^1/2^) and the peak current in a range from 25 to 500 mV s^−1^, so it can be inferred that the electro-oxidation processes are controlled by diffusion [4,15,23,24,25,26,27,28,29,30].

### 2.3. Effect of CB+IL on the PGE Performance

**Figure 3:**
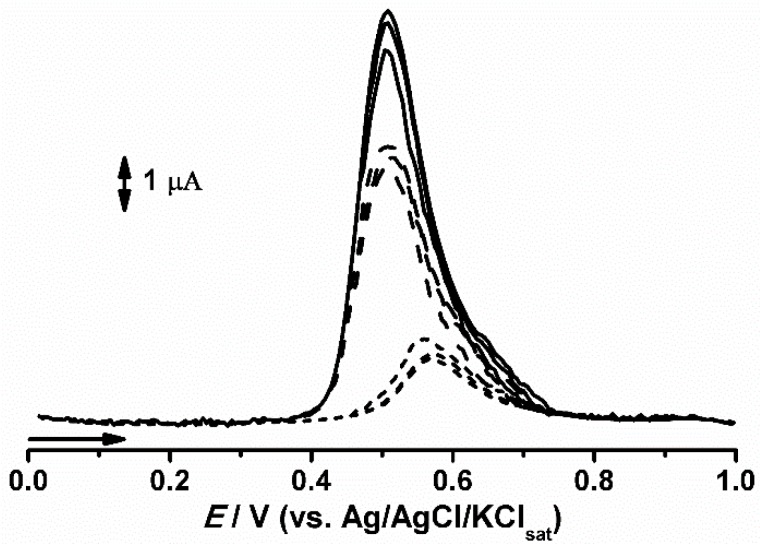
DPVs performed with bare PGE (**----**), IL/PGE (– – –), and CB+IL/PGE (–––) for 40 µmol L^−1^ DIC in pH 3.0 0.1 mol L^−1^ ACS (n = 3).

Owing to the recognized properties of CB [14,15,16,17,18,19], its effect as a modifying agent was investigated focusing on the possibility to extend the applications of PGE for samples containing low DIC levels. Concerning the feasibility of the manufacturing procedures, the approach herein used was easily conducted and consisted of an adsorption/immobilization method. In this context, the CB was suspended in IL in equal proportion. The choice for IL was due to its good conducting property and low proneness to undergo lixiviation process. Figure 3 shows DPV responses of bare PGE and two modified electrodes, namely IL/PGE and CB+IL/PGE (Figure 3).

It can be observed that the analytical signal had a notable improvement in both IL/PGE and CB+IL/PGE when compared to bare PGE (Figure 3). The increase of peak amplitude in IL/PGE could have occurred due to favorable molecular interactions between IL (1-butyl-3-methylimidazolium) and DIC, as DIC chemical structure is prone to allow van der Waals, electrostatic, and ionic interactions. Therefore, IL modified PGE signal enhancement is exerted by a pre-accumulation effect. However, a greater increase in the signal was observed in the CB+IL/PGE, due to the superior increment of the electrode surface area promoted by the CB nanostructured system [27,28,29].

### 2.4. Quantitative Determination of DIC in Pharmaceutical Samples

**Figure 4:**
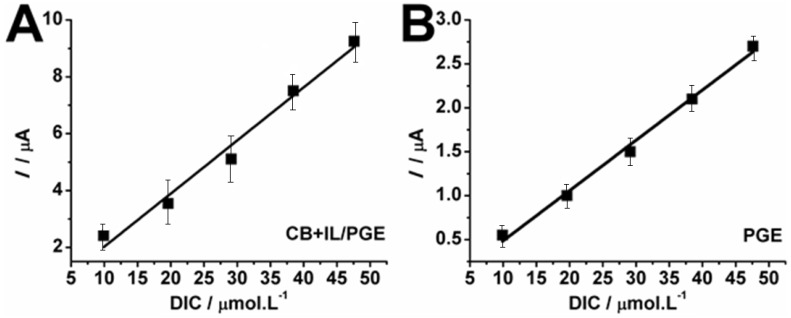
Calibration curve obtained from DPV assays for increasing DIC concentrations at CB+IL/PGE (**A**) and PGE (**B**) in pH 3.0 0.1 mol L^−1^ ACS.

To verify the suitability of the DPV assay using PGE and CB+IL/PGE for quantitative determinations of DIC in commercial drug tablets, calibration curves were conducted in increasing concentrations (Figure 4).

The resulting linear regression equations were: y = 1.5410 × 10^−7^ (±0.0098) + 1.8605 × 10^−7^ × (r = 0.9986) and y = 8.6102 × 10^−8^ + 5.7208 × 10^−8^ × (r = 0.9922) and, whereas the relative standard deviation values (RSD, n = 3) ranged from 3 to 8% and 2 to 5% for CB+IL/PGE and PGE, respectively. No calibration curve was constructed for IL/PGE as it was intended solely for comparative purposes concerning CB+IL/PGE sensibility.

The addition–recovery studies of DIC in tablet samples (n = 6) were performed for PGE, CB+IL/PGE, HPLC, and UV-Vis spectrophotometry. The DP voltammetric assays at PGE showed recovery of 98.4% with values close to other electroanalytical works [16,17,18,19]. RSD for this electrode was lower than the required value of 5% [2] and calculated limits of detection (LOD) and limits of quantification (LOQ) were respectively of 0.27 µmol L^−1^ and 0.91 µmol L^−1^. CB+IL/PGE showed a recovery of 99.4% and displayed LOD of 0.08 µmol L^−1^ and LOQ of 0.28 µmol L^−1^. These results indicate that matrix effects do not affect PGE and CB+IL/PGE determination of DIC. The comparison between the obtained results using the proposed modified electrode and the official methods (HPLC and UV-Vis spectrophotometry) are presented in Table 1.

Table 2 presents the LOD and LOQ of this work compared to results obtained with data in the literature that used electrochemical techniques in the determination of DIC.

However, many works [34,35] have achieved impressively lower LOD; this feature is not imperative for quality control applications. Moreover, the pencil graphite and carbon black is much less expensive, whereas the cleanness and practical procedure to renew the electrode surface are very attractive [18].

To validate the results, a statistical test of t-Student and ANOVA were performed for the DIC determination values (Table 3) at the 0.05 significance level (α). The calculated values of t (−0.75) and F (0.17) were smaller than tabulated critical values; thus, the variance of the obtained results for all performed methods (PGE, CB+IL/PGE, HPLC, and UV-Vis spectrophotometry) are not statistically different.

### 2.5. Accelerated Stability Test of DIC in Pharmaceutical Samples

The CB+IL/PGE was also used to monitor the stability of DIC tablets. In this context, the three tablet lots (A, B, and C) were submitted to stress through incubation in a thermal stove with 40 °C ± 5.0 for 6 months (Table 4). Analytical assays were performed before incubation, after three months of incubation and after six months of incubation.

As expected, the recovery values of voltammetric and chromatographic determinations showed virtually no degradation of the samples in all analyzed lots in the three evaluated times (Table 4). All found concentrations were less than 5% of the relative standard deviation. Both methods displayed effective approaches to evaluate the stability of DIC tablets under thermal stress conditions. Moreover, no evidence of degradation products was found in neither approach.

### 2.6. Interference Study

**Figure 5:**
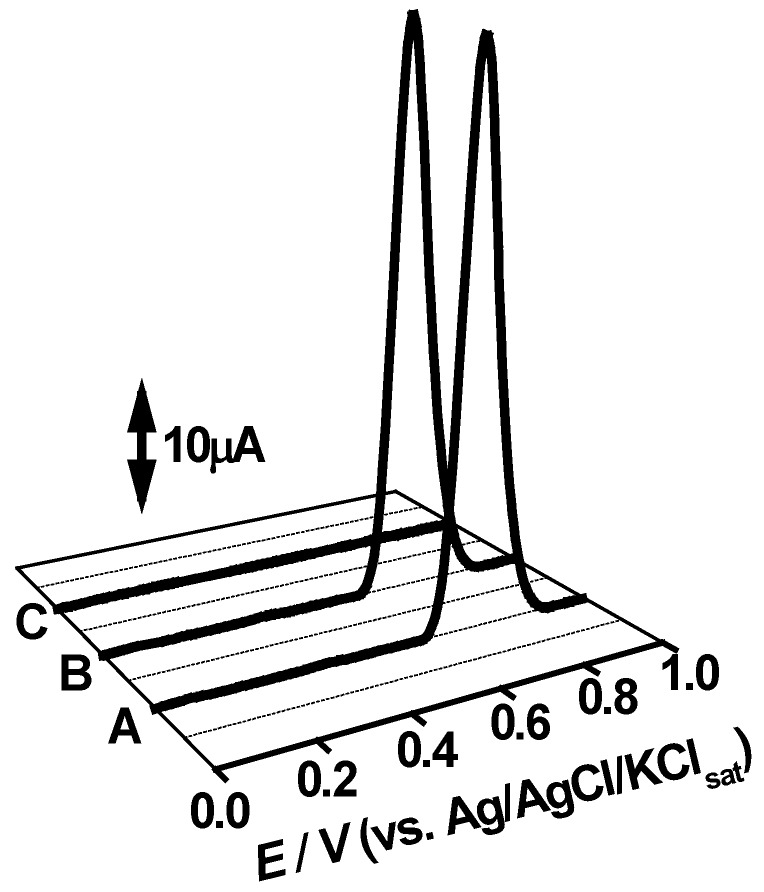
(**A**) DPV of 50 µmol L^−1^ DIC in pH 3.0, 0.1 mol L^−1^ ACS (n = 3) solution with 1 mg of manitol, lactose, and hydroxipropil cellulose; (**B**) 50 µmol L^−1^ DIC in pH 3.0 0.1 mol L^-1^ ACS (n = 3) solution; (**C**) 1 mg of manitol, lactose, and hydroxipropil cellulose in pH 3.0 0.1 mol L^−1^ ACS (n = 3) solution. All analysis conducted at CB+IL/PGE.

To evaluate the interference of different excipients in DIC determination, DPV analysis was conducted to ascertain their non-electro-activity. Results are displayed in Figure 5.

Results evidenced that all tested excipients did not display electro-activity, as corroborated by previous experiments done with DIC tablets. Moreover, excipients did not interfere with the signal gathering of DIC (Figure 5). Therefore, the results show that the method proposed in this work is nonetheless selective concerning DIC determination in pharmaceutical forms.

## 3. Materials and Methods

### 3.1. Reagents, Samples, and Solutions

The chemicals and solvents were of analytical grade and used without further purification. Analytical grade DIC potassium salt was purchased from Sigma (Saint Louis, MO, USA) and the stock solutions were prepared immediately before the experiments. Manitol, lactose, and hydroxipropil cellulose were donated by University Pharmacy of the Federal University of Goiás, and their stock solutions were prepared using the same protocol of DIC stock solutions. 1-Butyl-3-methylimidazolium hexafluorophosphate was purchased from Acros Organics, Geel, Belgium. 4B, 2.0 mm diameter graphite pencils were purchased from Koh-I-Hdmuth (Czech Republic). CB (Vulcan^®^ VXC72R, Cabot Corporation, Alpharetta, GO, USA) was kindly provided by Prof. Orlando Fatibello-Filho from UFSCar.

Electrolyte solutions were prepared by using high analytical grade salts, which were diluted in double distilled Milli-Q water (conductivity ≤ 0.1 µS cm^−1^) (Millipore S. A., Molsheim, France).

### 3.2. PGE Preparation

**Scheme 1:**
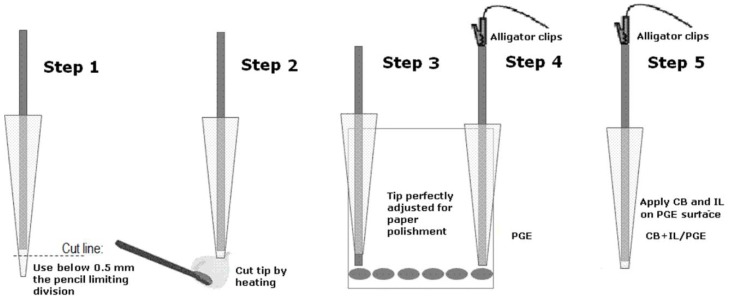
PGE and CB+IL/PGE preparation.

PGE was produced simply by fitting graphite pencil into a plastic tube c.a. 2.0 mm in such a way that the pencil was firmly placed and only 2.0 mm diameter circular area was in contact with the analytical solution (Scheme 1: steps 1 and 2). The pencil was polished in a Jet401 Norton 47F 1200 sandpaper after each assay and then further smoothed in writing paper. The polishment was achieved by drawing 6 small full circles of 0.5 cm, whereas smoothing was executed by doing 5 full circles of the same size. The electrical connection was made with alligator clips in the other extremity of the electrode surface/solution interface (Scheme 1).

### 3.3. CB+IL/PGE Preparation

To produce the CB+IL/PGE, a mixture of (1:1) CB powder and IL, 1-butyl-3-methylimidazolium hexafluorophosphate, was prepared. The mixture was rinsed thoroughly until a homogeneous paste was formed. A bare PGE, with only the circular surface area of the tip exposed, was smoothly pressed against the resulting paste resulting in a homogenous surface.

The effects of IL on PGE were also tested through DIC assessment using IL/PGE. The preparation of IL/PGE was performed by immersing PGE in IL for 15 min. IL/PGE was then dried at room temperature.

### 3.4. Electroanalytical Assays

Voltammetric experiments were carried out with a potentiostat/galvanostat µAutolab III^®^ integrated to the GPES 4.9^®^ software, Eco-Chemie, Utrecht, The Netherlands. The measurements were performed in a 5.0 mL one-compartment electrochemical cell, with a three-electrode system consisting of a PGE; IL/PGE or CB+IL/PGE, a Pt wire and a Ag/AgCl/KCl_sat_ (both purchased from Lab solutions, São Paulo, Brazil), representing the working electrode, the counter electrode and the reference electrode, respectively. The experimental conditions for differential pulse voltammetry (DPV) were: pulse amplitude 50 mV, pulse width 0.5 s, and scan rate 10 mV s^−1^. The experimental conditions for square wave voltammetry (SWV) were: pulse amplitude 50 mV, frequency 50 Hz, and a potential increment of 2 mV, corresponding to a scan rate of 100 mV s^−1^. The experimental conditions for cyclic voltammetry (CV) were: scan rate of 100 mV s^−1^ and scan range from 0 to 1 V, however, CV was also performed in different scan rates of 25, 50, 100, 250, 500 mV s^−1^. All experiments were done at room temperature (21 ± 1 °C) in sextuplicate (n = 6) and the main electrolytes used were 0.1 mol L^−1^ acetate (ACS) and phosphate buffer solutions (PBS), pH 3.0–9.0. The DP voltammograms were background-subtracted and baseline-corrected. All data were analyzed and treated with Origin 8^®^ software.

### 3.5. Determination of DIC in Tablets

To quantify DIC content in tablet form, an electroanalytical method and two official compendia approaches were chosen for comparative reasons, namely: spectrophotometry and HPLC assays. The analyzes using the official methods were performed according to the Brazilian pharmacopeia [2]. For electroanalytical assays, DIC stock solutions were prepared by weighing 20 tablets, then transferring enough powder in a 1 mmol L^−1^ NaOH solution to render 200 mL of 1 mmol L^−1^ DIC solution. Moreover, the resulting solution was sonicated at room temperature (22 ± 1 °C) for 20 min and then filtered in filter paper. The stock solution was prepared shortly before analysis, whereas the analytical measurement range was 5.0 to 500 µmol L^−1^.

The spectrophotometric measurements were carried out by using a UV–Vis spectrophotometer (Quimis, model Q-798U2VS, São Paulo-SP, Brazil). All samples were analyzed in a 1 cm length glassy cell at room temperature.

The chromatographic assays were performed in a Shimadzu HPLC system (Shimadzu, Kyoto, Japan) consisting of an LC-20AT pump, DGU-20A5 degasser, SPD-M20A PDA detector, CTO-20A column oven, and SIL-20A autosampler. The column oven temperature was set to 30 °C. The separation of the analytes was carried out using stainless steel ACE column packed with C18 (250 x 4.6 mm, 5 µm size particle). The mobile phase was a mixture of 50 mmol L^−1^ potassium phosphate buffer (pH 3.0) solution and methanol 80:20 v/v. The mobile phase was previously filtered before use through a 0.45 µm membrane (Millipore, Milford, MA, USA) and degassed in an ultrasonic bath (Unique USC-2800) for 20 min. The flow rate was of 1.0 mL min^−1^. The detection was performed at a specific wavelength of 275 nm and the data obtained for external standards and samples were processed and compared by LC Solutions software. In these conditions, the retention time of DIC was 9.2 min.

## 4. Conclusions

CB+IL/PGE compared to other sensors and assessment methods offers a satisfactory analytical performance for DIC determination in different pharmaceutical samples. Such a feature, when associated with low cost, easy access, quick, and efficient cleaning of the electrode area, indicates that PGE may be a useful tool for DIC analysis. Moreover, both PGE and its modified counterpart exhibit satisfactory detection and recovery, although standard deviation values were slightly higher than most of the sensors and methods applied. Nevertheless, results are in accordance with the specifications for such analysis.

CB+IL/PGE evidenced superior signal in comparison with bare PGE. The overall analytical performance and the low material cost associated with the prompt analysis provided by both electrodes consistently justifies the choice of these analytical devices as alternative approaches for drug quality control.

## Figures and Tables

**Table 1:** Determination of DIC tablets of DPV with unmodified (PGE) and modified (CB+IL/PGE) electrodes, high-performance liquid chromatography (HPLC), and UV-Vis spectrophotometry and their respective standard deviations (n = 6).

Sample	Labeled (mg)	DPV-PGE (mg)	DPV-CB+IL/PGE (mg)	HPLC (mg)	UV-Vis Spectrometry (mg)
DIC tablets 1	70	68.9 ± 3.5	69.6 ± 2.8	69.6 ± 2.1	68.7 ± 3.1
DIC tablets 2	70	69.1 ± 3.4	69.0 ± 2.9	69.8 ± 2.2	68.8 ± 3.0

**Table 2:** Comparisons of the limits of detection (LOD) and the limits of quantification (LOQ) in the determination of DIC with electroanalysis.

Electrode	Method	LOD (µmol L^−1^)	LOQ (µmol L^−1^)	Reference
CB+IL/PGE	DPV	0.08	0.28	Our work
Platinum Disk	LSV	5.4	16.2	[29]
IL/CNTPE	SWV	0.09	-	[27]
CPE-MWCT	DPV	0.74	3.49	[30]
Carbon paste/CNT’s	DPV	0.9	2.96	[31]
CuZEGE^g^	DPV	0.3	-	[32]
IL/CNTPE	DPV	0.2	-	[33]
MWCNT-IL	DPV	0.0003	-	[34]
F–CNF	SWV	0.0001	-	[35]

**Table 3:** Recovery values of DIC tablet with voltammetric and chromatographic assays of the three different industrial lots.

t Student *		ANOVA *	
t value	−0.7513	F value	0.1796
Prob. > t	0.4863	Prob. > F	0.6806

* α = 0.05 (significance level).

**Table 4:** Recovery values of DIC tablet with voltammetric and chromatographic assays of three different industrial lots.

		Found Recovery (% ± RSD)	
Lot	Months	Voltammetry	Chromatography
**A**	0′	99.9% ± 2.0%	100.2% ± 0.72%
	3′	100.5% ± 2.2%	100.4% ± 1.2%
	6′	101.3% ± 1.5%	100.9% ± 1.0%
**B**	0′	101.4% ± 2.1%	102.0% ± 1.0%
	3′	100.7% ± 2.3%	101.2% ± 1.5%
	6′	100.9% ± 1.8%	101.6% ± 1.1%
**C**	0′	101.9% ± 2.1%	101.7% ± 1.2%
	3′	100.7% ± 2.7%	101.0% ± 1.6%
	6′	101.2% ± 1.9%	101.6% ± 0.89%
